# Supplementary material for: Metabolite Profiling and Biological Activity Assessment of Paeonia ostii Anthers and Pollen Using UPLC-QTOF-MS
Source: Int J Mol Sci. 2024 May 17;25(10):5462. doi: 10.3390/ijms25105462 (PMC11121493; doi:10.3390/ijms25105462)
Supplement: Supplementary file 1 [file ijms-25-05462-s001.zip › Supplementary materials S2.pdf]

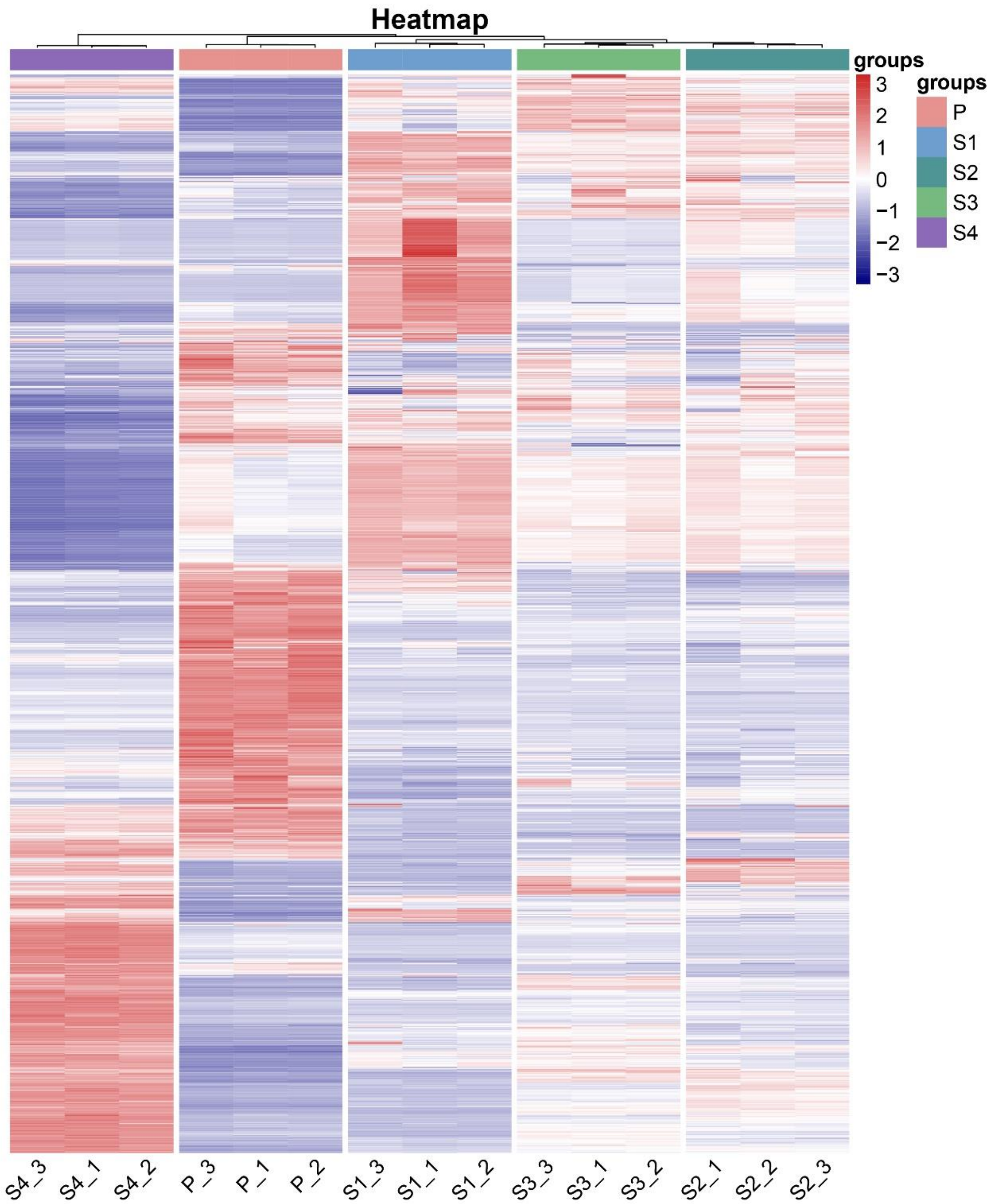

Figure S1. Hierarchical analysis (HCA) heat map of all detected ion peaks in different samples. Red and blue indicate higher and lower abundances, respectively.

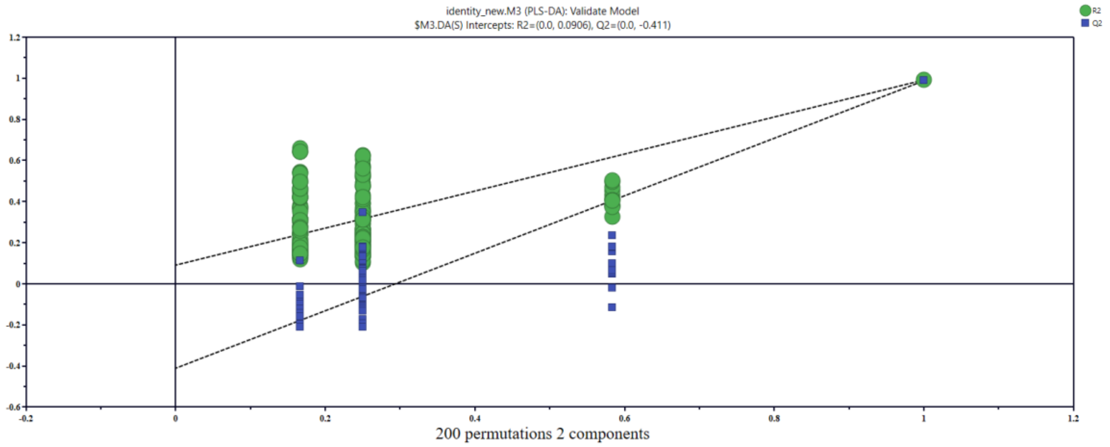

Figure S2. The permutation test plot of PLS-DA.
